# Network analysis of multidimensional symptom experience among postoperative esophageal cancer survivors

**DOI:** 10.1186/s12955-025-02459-8

**Published:** 2025-12-03

**Authors:** Yanfei Wang, Yingtao Meng, Xiaotong Li, Fang Zhang, Wenya Su, Ruixue Han, Junyi Peng, Miao Zhang, Shengfen Li, Ge Wang, Meimei Shang

**Affiliations:** 1 School of Nursing, Shandong First Medical University and Shandong Academy of Medical Sciences, Jinan, China; 2 Nursing Department, Shandong Cancer Hospital and Institute, Shandong First Medical University and Shandong Academy of Medical Sciences, Jinan, China; 3 Ordos central hospital, Ordos, China; 4 Esophageal Surgical Department, Shandong Cancer Hospital and Institute, Shandong First Medical University and Shandong Academy of Medical Sciences, Jinan, China; 5 School of Nursing, Shandong University of Traditional Chinese Medicine, Jinan, China; 6 Qingzhou Wangfu Health Center, Weifang, China

**Keywords:** Esophageal cancer, Surgery, Multidimensional symptoms, Network analysis

## Abstract

**Background:**

Postoperative symptom burden is considerable and markedly undermines the quality of life of esophageal cancer (EC) survivors. This study aimed to examine symptom clusters and the interrelationships among symptoms in postoperative EC survivors, with the goal of identifying core symptoms.

**Methods:**

A cross-sectional study was conducted using the European Cancer Life Questionnaire and the EC-Specific Supplementary Questionnaire. EC survivors were recruited in Shandong between February 2023 and February 2024. Principal component analysis (PCA) was utilized to identify symptom clusters, while Gaussian graphical network models were used to estimate the network structure.

**Results:**

A total of 460 EC survivors were included in the study, revealing three distinct symptom clusters: the reflux-dysphagia cluster, the respiratory-related symptom cluster, and the recovery-fatigue cluster. The final network model demonstrated interconnections among these symptoms. “Fatigue” (FA) exhibited the highest strength centrality, identifying it as the most prominent core symptom in the network. “Emotional functioning” (EF), “Fatigue” (FA), and “cognitive functioning” (CF) ranked highest in terms of bridge strengths. Additionally, the model showed excellent network stability.

**Conclusions:**

EC survivors experienced significant postoperative symptom burden, with symptom network analysis revealing the complex interrelations among postoperative symptoms. This approach also identified core symptoms that play a crucial role in the network. Fatigue emerged as the most influential core symptom, highlighting the significance of targeted interventions to mitigate negative symptom interactions and improve quality of life.

## Introduction

In 2022, data from the International Agency for Research on Cancer indicated that China contributed 43.8% of the global incidence and 42.1% of the global mortality of esophageal cancer (EC) [1]. Despite the downward trend in age-standardized incidence and mortality rates in recent years, the burden of EC in China continues to rank among the highest globally [2]. Surgery remains the primary treatment for EC [3]. Nevertheless, esophagectomy remains a highly challenging procedure [4], and survivors frequently suffer from symptoms such as pain, fatigue, chest discomfort and reflux. These postoperative manifestations may adversely influence recovery and ultimately affect long-term prognosis [5, 6]. It is essential to improve the postoperative quality of life for EC survivors by identifying appropriate and effective symptom management strategies [7]. Symptom clusters, defined as groups of interrelated postoperative symptoms that exhibit synergistic effects [8], can substantially exacerbate survivors’ psychological burden, compromise treatment adherence and adversely affect postoperative recovery [9]. Precision nursing entails creating personalized interventions based on the specific needs of each survivor [10]. However, due to the complexity of postoperative symptom clusters, traditional interventions that address only individual symptoms are no longer sufficient. These symptoms frequently present as interrelated clusters that mutually reinforce one another and adversely influence outcomes such as physical functioning, emotional well-being and social reintegration, thereby intensifying the challenges of survivors’ recovery. Therefore, understanding the mechanisms underlying their interactions and identifying core symptoms are critical for predicting quality of life and informing targeted interventions [11]. Network analysis offers a robust methodological framework for this purpose by modeling the complex interrelationships among symptoms.

Network analysis constructs a model composed of “nodes” and “edges” providing visual representations that highlight the importance of each variable and the complex interconnections within the network [12]. Within this framework, centrality indices are used to quantify the relationships among symptoms and to identify the most influential, or “core” symptoms. This analytical approach has been widely applied to explore the dynamic interactions between psychological and chronic disease–related symptoms. Previous research has applied network analysis to examine the interrelationships among anxiety, depression and fear of cancer recurrence in breast cancer survivors [13]. Similarly, studies involving elderly survivors with advanced cancer have employed network analysis to demonstrate that anxiety and depression are closely linked to the overall symptom burden [14]. Furthermore, cross-lagged network analysis has been employed to examine the interrelationships among symptoms across different postoperative stages in colorectal cancer survivors, revealing that persistent postoperative pain exerts a detrimental effect on recovery [15].

With the advancement of personalized medicine, there is growing recognition that managing individual symptoms alone is insufficient to address the complex challenges that arise after surgery. Consequently, symptom-based systematic analyses have gained increasing importance in recent years. Network analysis offers a novel approach to identifying core symptoms and elucidating the intricate structure of symptom clusters by visualizing and quantitatively characterizing the interrelationships among symptoms [16]. The early identification of key postoperative symptoms may provide a scientific basis for developing comprehensive and multidimensional symptom management strategies. Such efforts are expected to enhance the quality of life of EC survivors.

## Methods

### Study design and ethical compliance

This cross-sectional study evaluated postoperative symptoms among survivors who underwent radical resection of EC in a tertiary cancer hospital in the north of China between February 2023 and February 2024. Ethical approval was obtained from the Institutional Ethics Committee (Approval No. SDTHEC2023002004). All procedures were conducted in accordance with the principles of the Declaration of Helsinki, and written informed consent was obtained from all participants prior to data collection.

### Eligibility criteria and participants

Survivors who underwent radical resection of EC were included as study participants. Inclusion criteria: Participants were required to be over 18 years old, have a histologically confirmed diagnosis of EC with radical resection, be cognitively intact with a stable mental state and provide voluntary informed consent. Exclusion criteria: Individuals were excluded if they were unconscious, had trouble communicating, experienced cognitive impairment, had malignancies in other organs, or were already taking part in other clinical trials.

The sample size was determined using the cross-sectional formula N = 50 + 8 × n, where n represents the number of independent variables [17]. Given that there were 35 independent variables and considering a 20% attrition rate, a minimum of 396 participants was required. Moreover, previous studies have suggested that a sample size exceeding 300 was generally considered adequate for network analysis to ensure result reliability [18].

### Symptom assessment and data collection

#### Demographic and clinical characteristics

The study was designed in accordance with the research objectives and a comprehensive review of relevant literature. The collected data encompassed general demographic characteristics (age, sex, height, weight, educational level, marital and employment status, and smoking or drinking habits) as well as disease-related variables such as pathological type.

#### European organization for research and treatment of cancer quality of life questionnaire (EORTC QLQ-C30)

The EORTC QLQ-C30, developed by the European Organization for Research and Treatment of Cancer (EORTC), is a widely used instrument for assessing the quality of life in cancer survivors. It is applicable to patients with all cancer types. The questionnaire comprises 30 items organized into 15 domains and employs a 4-point Likert scale with response options ranging from “not at all” to “very much” (scored from 1 to 4). Items 29 and 30 are rated on a 7-point scale (1–7). A higher score in the overall health and functioning domains indicates better quality of life, while a lower score in the symptom domains indicates better quality of life [19–21]. The Chinese version of the QLQ-C30 has demonstrated good psychometric properties, with Cronbach’s α coefficients ranging from 0.75 to 0.88 and test–retest reliability coefficients ranging from 0.75 to 0.89 [22].

#### European organization for research and treatment of EC survivor supplementary questionnaire (EORTC QLQ-OES18)

The QLQ-OES18 is a supplementary module specifically developed for EC to assess disease-specific symptoms, as well as treatment-related effects and side effects. It comprises 18 items, organized into four multi-item subscales and six single-item measures. All items are rated on a 4-point Likert scale, with higher scores reflecting a greater symptom burden. The Cronbach’s α coefficients for the multi-item subscales range from 0.689 to 0.822 [23], indicating satisfactory reliability and validity.

### Statistical analysis and outcome measures

Descriptive statistical analyses of survivors’ demographic characteristics were performed using SPSS version 27.0. Quantitative variables with a normal distribution were expressed as the mean ± standard deviation (SD), whereas non-normally distributed variables were presented as the median and interquartile range (IQR). Categorical variables were summarized as frequencies and percentages. Exploratory factor analysis was performed on postoperative single symptoms, using principal component analysis to extract common factors, followed by varimax orthogonal rotation for factor rotation [24]. Factors were retained if their eigenvalues were ≥ 1 and factor loadings were ≥ 0.5 [25].

Network analysis was performed using the bootnet and qgraph packages in R version 4.4.2. The correlation matrix was first optimized and regularized using the EBICglasso algorithm, followed by visualization of the network structure with the qgraph function. In the network, nodes represented individual symptoms, while edges reflected the associations between them. The thickness of each edge indicated the strength of the correlation between two symptoms. Community detection was conducted using the Louvain algorithm, with edge weights serving as the primary indicators of connection strength. Network centrality indices—including strength, closeness, and betweenness—were calculated to quantify the importance of each symptom within the network [26]. The accuracy and stability of the network were assessed using a bootstrapping approach. The correlation stability (CS) coefficient was computed to evaluate the robustness of centrality indices; according to previous literature, a CS value greater than 0.25 was considered acceptable, and values above 0.5 were regarded as ideal [27]. Finally, a nonparametric bootstrap method was applied to estimate the accuracy of edge weights based on 95% confidence intervals (CIs), as implemented in the bootnet package. Narrower CIs indicated higher accuracy and greater reliability of the network structure [27].

## Results

### Demographic characteristics and clinical features

A total of 460 participants were included in this study. Among them, 315 participants (68.5%) were 60 years or above. 81.7% were male, as presented in Table 1.

**Table 1:** General information of patients [n(%)]

Variables	N = 460
**Age (years)**	63.64 ± 4.24
<60	145 (31.5)
≥60	315 (68.5)
**Gender**	
Male	376 (81.7)
Female	84 (18.3)
**Marital status**	
Married	448 (97.4)
Single or divorced	12 (2.6)
**Educational level**	
Primary school and below	160 (34.8)
Junior high school	189 (41.1)
Senior high school	91 (19.8)
University	20 (4.3)
**Occupation**	
Farmer	292(63.5)
Not employed/be on sick leave	88(19.1)
Other	80 (17.4)
**Smoking**	
Yes	198 (43)
No	262 (57)
**Alcohol**	
Yes	238 (51.7)
No	222 (48.3)
**Pathological type**	
Squamous Cell Carcinoma	387 (84.1)
Adenocarcinoma	73 (15.9)
**Primary caregiver**	
Spouse	200 (43.5)
Children	260 (56.5)
**Surgical approach**	
Minimally invasive	255 (55.4)
Open	205 (44.6)

### Network analysis results

#### Network structure

**Fig. 1:**
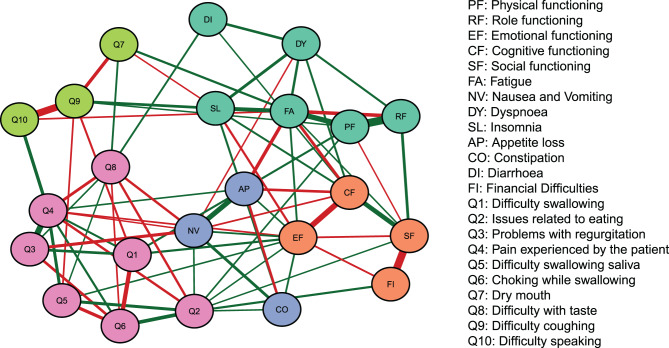
Network analysis of postoperative EC symptoms. Green lines represent positive correlations, while red lines represent negative correlations. The thickness of the edges indicates the strength of connectivity

**Fig. 2:**
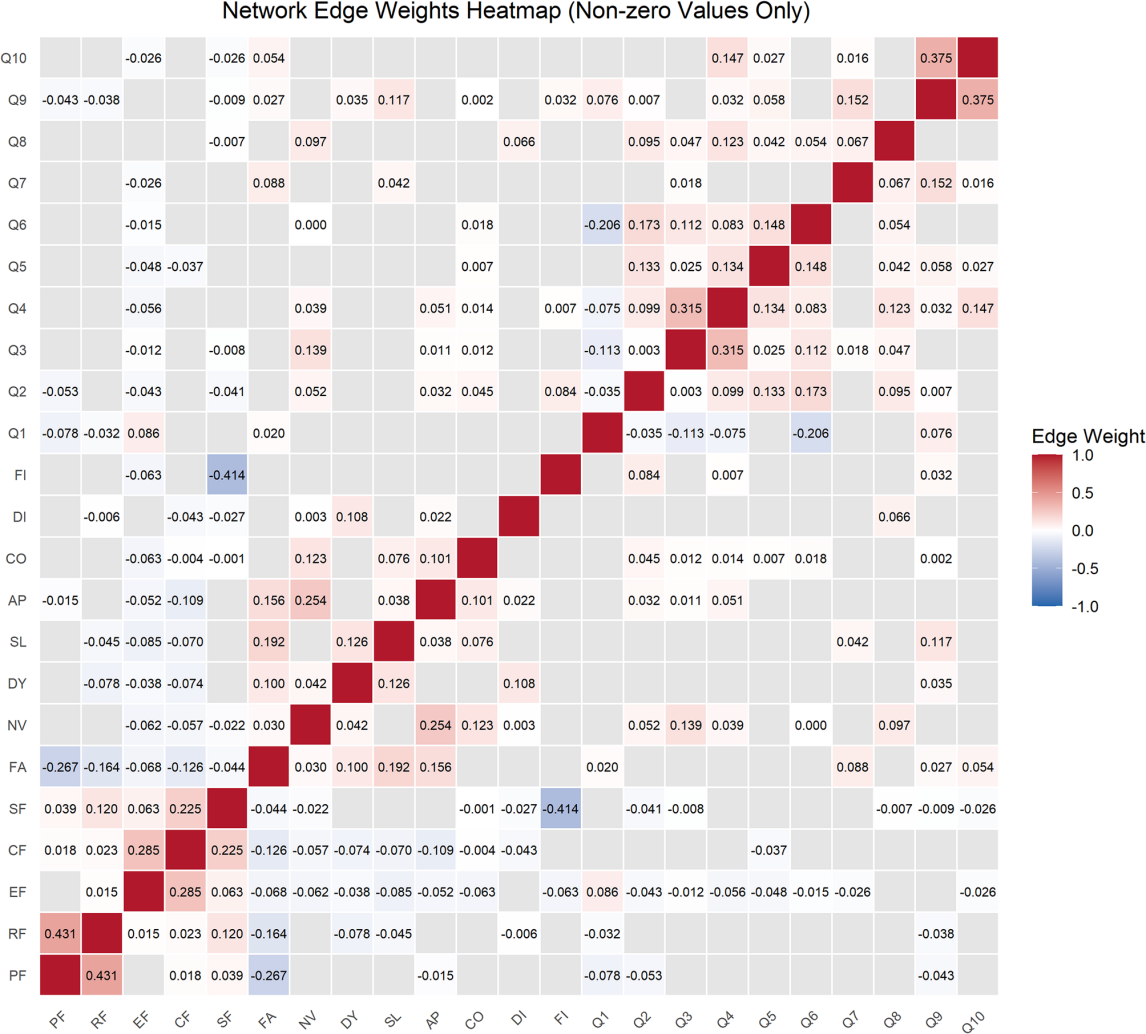
Heatmap depicting all non-zero edge weights

In this study, the duplicated symptom of pain appearing in both the QLQ-C30 and QLQ-OES18 and the overall quality of life score were excluded, resulting 23 symptoms for network analysis. The constructed network comprised 253 edges, of which 122 (48.2%) were estimated to be non-zero. To improve visualization, only the top 30% of edges by weight were retained for network plotting (Fig. 1), while the complete distribution of edge weights was shown in Fig. 2. Furthermore, based on edge weights, the network was partitioned into five clustered communities, with nodes within each community assigned identical colors. These communities may represent potential symptom clusters.

The strongest positive correlation was observed between role functioning and physical functioning (RF–PF, edge weight = 0.431), whereas the relationship between social functioning and financial difficulties (SF–FI, edge weight = –0.414) represented the strongest negative correlation. Additionally, notable positive associations were observed between cough difficulty with speech impairment (Q9–Q10, edge strength = 0.375), reflux with pain (Q3–Q4, edge strength = 0.315), and emotional functioning with cognitive functioning (EF–CF, edge strength = 0.285).

#### Centralization index and bridge strength

**Fig. 3:**
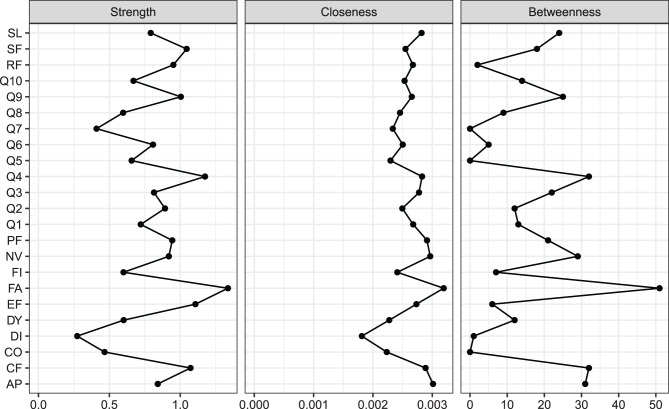
Centrality indices of the network: strength, closeness, and betweenness

**Fig. 4:**
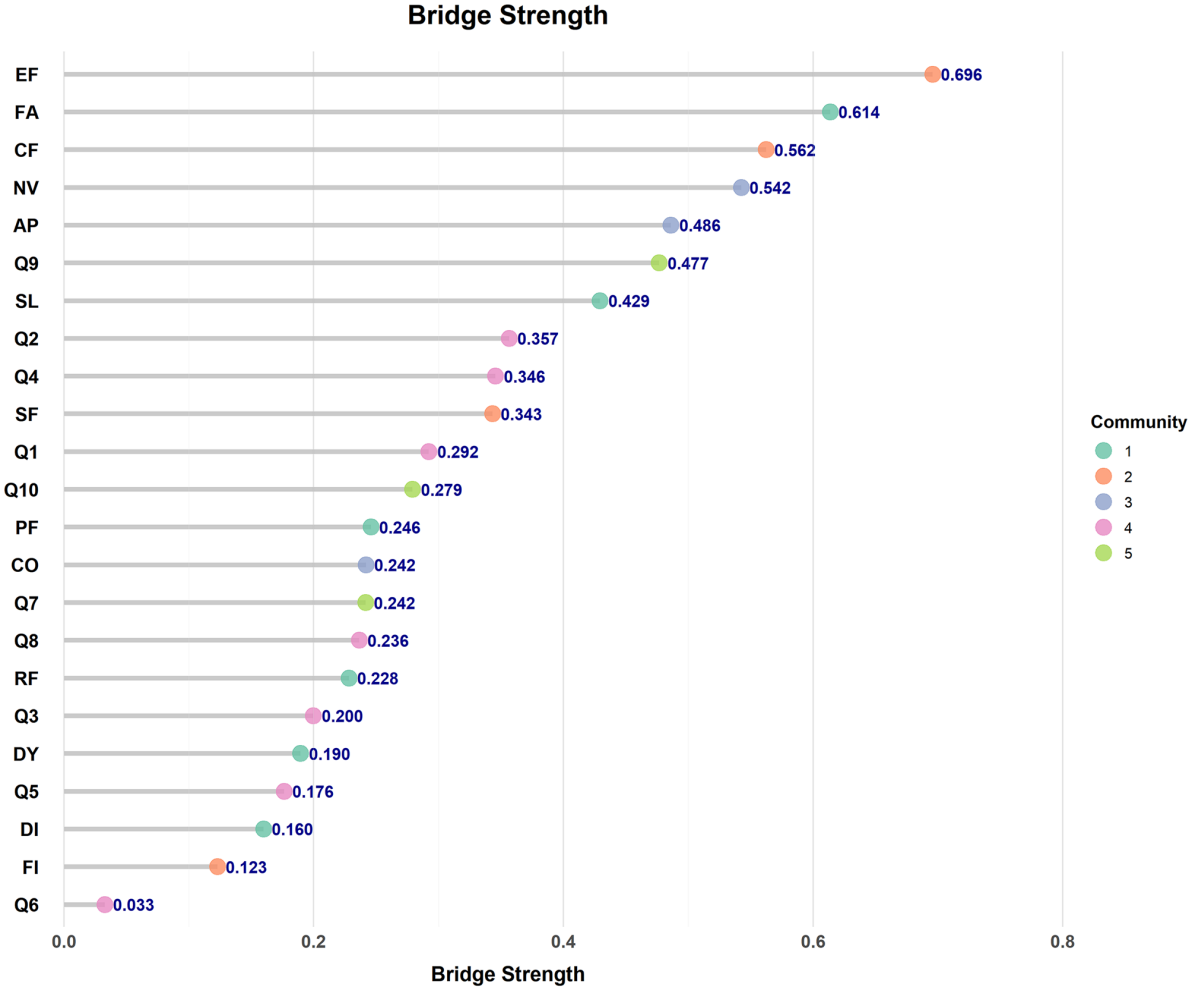
Bridge strength estimates in the symptom network

The strength, closeness, and betweenness centrality scores were presented in Fig. 3. Overall, fatigue (FA) exhibited the highest strength, closeness and betweenness centrality values, suggesting that this symptom was highly interconnected with other nodes and played a pivotal role within the network. The presence of fatigue exerted a substantial influence on the overall network structure. In contrast, diarrhea (DI) showed the lowest strength and closeness centrality scores. As illustrated in Fig. 4, the bridge strengths of emotional functioning (EF), fatigue (FA) and cognitive functioning (CF) were among the highest, indicating that these nodes act as crucial bridging symptoms linking distinct symptom clusters in the network.

#### The stability of the centralization index

**Fig. 5:**
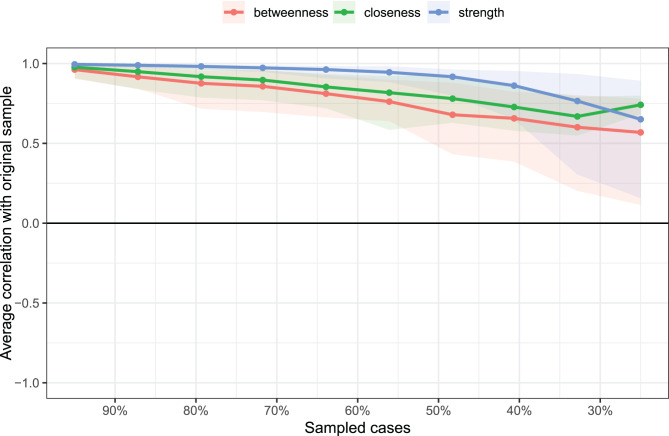
The stability of the centralization index

Fig. 5 presented the results of the centrality index stability. As the percentage of samples included in the estimation decreases (as indicated on the X-axis, with subset samples ranging from 95% to 25% of the original sample), the correlation between the estimates from the subset samples and the original full sample slowly declines, with the closeness estimates showing a slight rebound after the gradual decrease. Even when the subset sample size was reduced to 25% of the original, the estimated centrality indices for strength, betweenness, and closeness still remained above 0.5.

#### The accuracy of edge weights

**Fig. 6:**
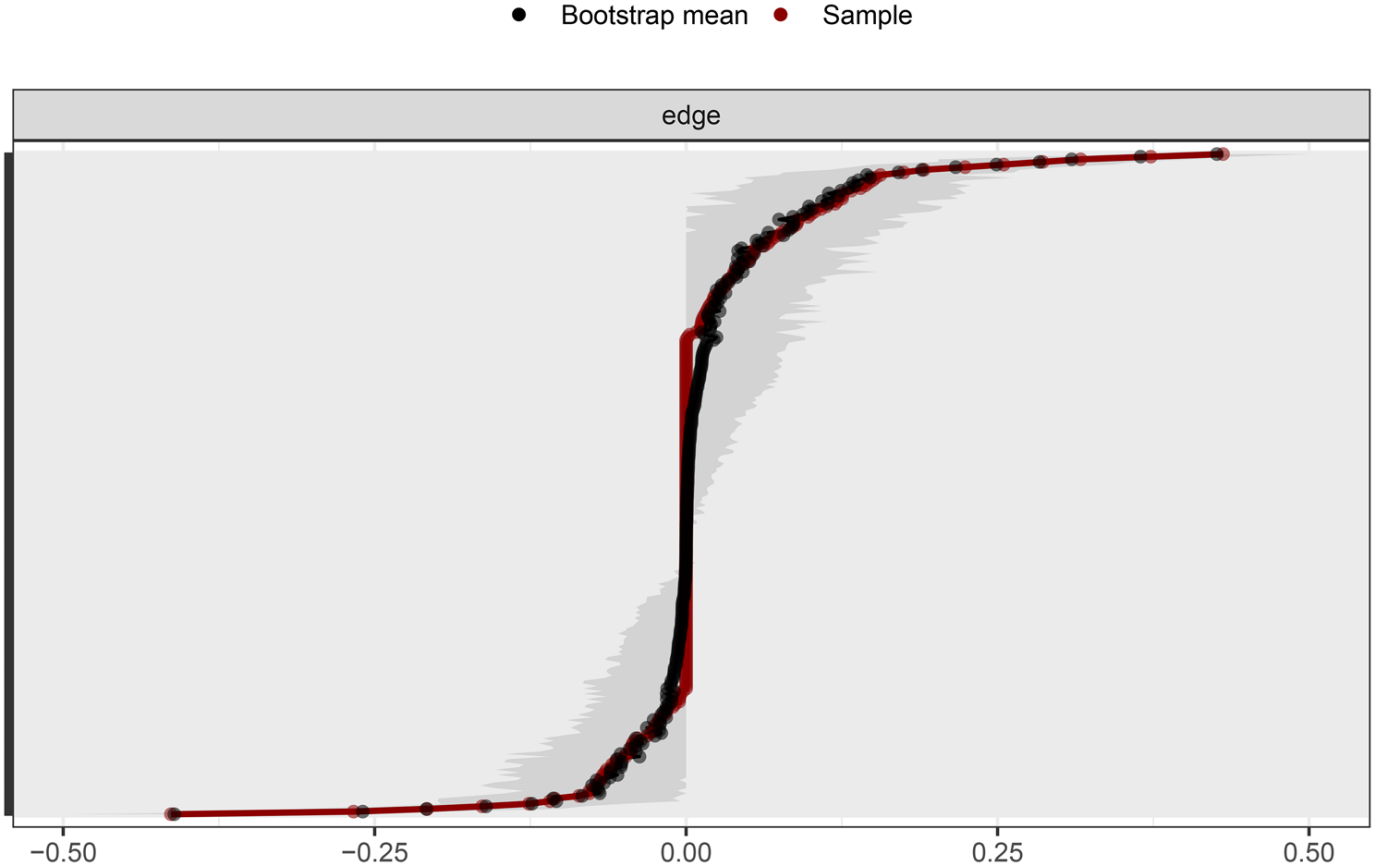
The accuracy of edge weight estimation (red line) and the 95% confidence interval of the estimation (gray bar)

The bootstrap CI for the edges was shown in Fig. 6. Based on the bootstrap CI of the edge weights, the gray areas represented the estimated 95% confidence intervals. Overall, these intervals were relatively narrow, indicating that the estimates were highly accurate.

### Symptom cluster extraction results

Building upon the network analysis, this study further focused on individual symptom patterns. Consistent with previous research, items pertaining to functional and economic dimensions were excluded, while symptoms with an incidence rate of ≥ 15% were retained for factor analysis [28]. The Kaiser–Meyer–Olkin value was 0.846, and Bartlett’s test of sphericity was significant (χ² = 1949.302, P < 0.001), indicating good sampling adequacy and suitability for factor analysis. The exploratory factor analysis extracted four factors with eigenvalues greater than 1, collectively explaining 53.13% of the total variance, as summarized in Table 2. Factors containing fewer than three items within the same component or with loadings < 0.50 were excluded. According to the characteristics of the symptoms within each factor, the groups were identified as the reflux and swallowing disorder group, the restorative fatigue symptom group, and the respiratory-related symptom group.

**Table 2:** The rotated component matrix

Symptom	1	2	3	4
Q1	−0.629			
Q2	0.591			
Q3	0.674			
Q4	0.689			
Q5	0.530			
Q6	0.712			
Q8	0.502			
FA		0.623		
SL		0.546		
AP		0.725		
NV		0.636		
CO		0.680		
Q7			0.545	
Q9			0.818	
Q10			0.737	
DI				0.826
DY				
Initial eigenvalue	4.552	2.077	1.317	1.086
Eigenvalues after rotation	2.969	2.394	2.299	1.370
Cumulative variance contribution rate (%)	17.467	31.549	45.072	53.131

## Discussion

In the network analysis graph, fatigue emerged as the symptom with the highest strength, closeness, and betweenness in the entire network. Strength reflects the sum of absolute edge weights between a node and its directly connected nodes, and a higher value indicates greater importance of that symptom within the network [12]. Fatigue isa persistent and subjective sense of exhaustion that affects physical, emotional and cognitive functioning, and is difficult to relieve through rest or sleep. Many survivors may also suffer from cancer-related fatigue, further compromising both physical and psychological well-being [29, 30]. Preoperative neoadjuvant therapy and surgical procedures may impair mitochondrial structure and disrupt mitochondrial energy metabolism, thereby reducing somatic function and aggravating fatigue [31]. Moreover, postoperative inflammatory responses and pain-related stress mayfurther exacerbate fatigue in these patients [32].

Previous studies on symptom management have primarily focused on commonly observed postoperative symptoms, often overlooking core symptoms that exert substantial influence on disease progression. Decreased appetite and pain, for example, are frequently reported among postoperative survivors [33, 34]. However, this study found that although symptoms like insomnia, pain, loss of appetite, dry mouth, and speech impairment cause significant distress to survivors, they did not emerge as core symptoms in the network analysis. Additionally, research has shown that the most prevalent symptoms among cancer survivors are not necessarily the core symptoms within the symptom network [35]. Core symptoms are more likely to exacerbate or trigger other symptoms.This study suggests that fatigue is a core symptom in postoperative EC survivors. However, in a study by Chen et al. on postoperative EC survivors, the core symptoms were acid indigestion or heartburn [36]. This discrepancy may be attributed to differences in patient demographics, including the older age of participants in the present study and the higher proportion of survivors who underwent thoracotomy. Therefore, this study highlights the need to reconsider clinical intervention priorities and to develop more targeted interventions. Since fatigue is a core symptom in this network, a comprehensive approach that includes cognitive therapy, physical activity and medication should be considered. These interventions may help improve overall symptom management for EC survivors [37].

Bridge symptoms refer to key symptoms that connect different symptom clusters, and their role can be evaluated through bridge strength [38]. Bridge strength is defined as the sum of the edge weights connecting a specific symptom directly to distinct symptom clusters [39], it is crucial for understanding the complex interrelationships among symptoms. In this study, emotional functioning, fatigue, and cognitive functioning exhibited the highest bridge strengths, indicating that they were the core bridge symptoms within the postoperative symptom network of EC. Castro D et al. suggested that bridge symptoms play a transmittal role within the network, making them potential therapeutic targets for interrupting symptom interactions [38]. Studies has shown that addressing bridge symptoms, such as sleep disorders, may mitigate the interconnected effects of depression and anxiety, potentially contributing to a reduction in overall symptom burden among survivors [40]. Therefore, in clinical practice, bridge symptoms are essential for intervention strategies. Targeted interventions focused on bridge symptoms have the potential to disrupt negative feedback loops between symptom clusters and enhance overall patient outcomes.

Emotional functioning, fatigue, and cognitive functioning were identified as key bridge symptoms in the postoperative symptom network of EC survivors. Among them, emotional functioning exhibited the highest bridge strength, indicating that healthcare providers should prioritize targeted psychological assessment and intervention. Previous studies have demonstrated that depression is an independent predictor of poor prognosis in EC survivors [41]. Changes in social functioning and related domains may further exacerbate psychological stress [42]. Moreover, prolonged psychological burden and stress have been associated with dysregulation of neural polyglutamic acid metabolism, which may upregulate inflammation and suppress protective immune responses, ultimately affecting immune function in cancer survivors [43]. Behavioral activation has emerged as a promising intervention, as it canreduce psychological distress not only directly, but also indirectly by enhancing self-management capacity as a mediating mechanism [44]. Additionally, behavioral activation can reduce stigma and significantly improve quality of life [45]. High-quality nursing care that integrates pain control, psychological support, and nutrition management has also been shown to substantially alleviate depressive symptoms [46]. Fatigue demonstrated a bridge strength index of 0.614. It was not only a central symptom within the overall symptom network but also played a critical role in linking distinct symptom clusters. Evidence indicates that increased postoperative fatigue in EC survivors may elevate the risk of postoperative complications [47]. Therefore, targeted fatigue interventions are essential. Multidisciplinary strategies, including psychological intervention, nutritional support, and sleep management, have proven effective in reducing fatigue in clinical nursing practice [48]. In addition, neoadjuvant therapy is widely recommended prior to esophagectomy [49], which may induce fatigue even before surgery. These findings highlight the necessity of preoperative fatigue assessment and the timely implementation of tailored interventions. Consistent with previous evidence, strong interconnections among different symptom clusters may further amplify adverse postoperative outcomes [50]. Thus, greater clinical emphasis should be placed on symptoms with high bridge strength to optimize postoperative recovery and improve quality of life among EC survivors.

At the level of individual symptoms among postoperative EC survivors, which could be categorized into three major clusters: the reflux and swallowing disorder symptom cluster, the respiratory-related symptom cluster, and the restorative fatigue symptom cluster. The reflux and swallowing disorder symptom cluster included symptoms like dysphagia, reflux, pain, throat clearing, and the feeling of food sticking in the throat. The respiratory-related symptom cluster comprised dry mouth, difficulty coughing, and speech impairment. Lastly, the restorative fatigue symptom cluster encompassed symptoms such as fatigue, insomnia, loss of appetite, and constipation. Research has shown that these symptom clusters interact with one another, leading to a vicious cycle among them. Previous studies have shown that EC survivors often experience severe reflux after surgery, which may significantly diminish their quality of life [51]. In postoperative survivors of EC, reflux may allow gastric contents to enter the airway, which could lead to coughing and potentially increase the risk of aspiration pneumonia, possibly exacerbating respiratory - related symptoms [52]. The ongoing presence of reflux and coughing may contribute to increased fatigue in survivors [53]. It’s crucial to concentrate not just on presenting individual symptoms but also on understanding the interactions among different symptom clusters. This approach is essential for optimizing the comprehensive management of survivors through multidimensional interventions.

## Limitations

Although the current symptom network analysis provided valuable insights for early postoperative symptom management, several limitations should be noted. The network model is constructed based on associations among variables, however, such associations do not imply causality. As a result, the study was unable to evaluate how individual differences, such as age or surgical approach, might influence symptom clusters. In addition, the analysis focused exclusively on the early postoperative period. Future longitudinal studies may help elucidate the temporal dynamics of symptom networks and symptom clusters across different postoperative stages, thereby offering more dynamic guidance for clinical nursing practice.

## Conclusion

This study revealed the complex interactions within the postoperative symptom network of EC survivors. Fatigue was identified as the most central symptom, exerting the strongest influence across the network. Emotional functioning, fatigue, and cognitive functioning emerged as key bridge symptoms that connected different symptom clusters. Future symptom management may benefit from a network-oriented and multidimensional approach, in which precision interventions focus on disrupting critical symptom links to reduce overall symptom burden, promote recovery, and enhance quality of life.

## Data Availability

Data are available from the corresponding author upon reasonable request.
